# Evaluation of carboxamide-type synthetic cannabinoids as CB_1_/CB_2_ receptor agonists: difference between the enantiomers

**DOI:** 10.1007/s11419-017-0378-5

**Published:** 2017-08-04

**Authors:** Takahiro Doi, Takaomi Tagami, Akihiro Takeda, Akiko Asada, Yoshiyuki Sawabe

**Affiliations:** Division of Hygienic Chemistry, Osaka Institute of Public Health, 1-3-69 Nakamichi, Higashinari-ku, Osaka, 537-0025 Japan

**Keywords:** Carboxamide-type synthetic cannabinoid, Enantiomeric difference in activity, CB_1_/CB_2_ receptor agonist, AB-FUBINACA 2-fluorobenzyl isomer, APP-CHMINACA, 5F-EMB-PINACA

## Abstract

Recently, carboxamide-type synthetic cannabinoids have been distributed globally as new psychoactive substances (NPS). Some of these compounds possess asymmetric carbon, which is derived from an amide moiety composed of amino acid derivatives (i.e., amides or esters of amino acids). In this study, we synthesized both enantiomers of synthetic cannabinoids, *N*-(1-amino-3-methyl-1-oxobutan-2-yl)-1-(2-fluorobenzyl)-1*H*-indazole-3-carboxamide (AB-FUBINACA 2-fluorobenzyl isomer), *N*-(1-amino-1-oxo-3-phenylpropan-2-yl)-1-(cyclohexylmethyl)-1*H*-indazole-3-carboxamide (APP-CHMINACA), ethyl [1-(5-fluoropentyl)-1*H*-indazole-3-carbonyl]valinate (5F-EMB-PINACA), ethyl [1-(4-fluorobenzyl)-1*H*-indazole-3-carbonyl]valinate (EMB-FUBINACA), and methyl 2-[1-(4-fluorobenzyl)-1*H*-indole-3-carboxamido]-3,3-dimethylbutanoate (MDMB-FUBICA), which were reported as NPS found in Europe from 2014 to 2015, to evaluate their activities as CB_1_/CB_2_ receptor agonists. With the exception of (*R*) MDMB-FUBICA, all of the tested enantiomers were assumed to be agonists of both CB_1_ and CB_2_ receptors, and the EC_50_ values of the (*S*)-enantiomers for the CB_1_ receptors were about five times lower than those of (*R*)-enantiomers. (*R*) MDMB-FUBICA was shown to function as an agonist of the CB_2_ receptor, but lacks CB_1_ receptor activity. To the best of our knowledge, this is the first report to show that the (*R*)-enantiomers of the carboxamide-type synthetic cannabinoids have the potency to activate CB_1_ and CB_2_ receptors. The findings presented here shed light on the pharmacological properties of these carboxamide-type synthetic cannabinoids in forensic cases.

## Introduction

In recent years, several new psychoactive substances (NPS) have been newly identified from illicit drug products, one after another, and have been abused as legal alternatives to scheduled drugs [1]. One of the most popular classes of NPS is synthetic cannabinoids, which are recreationally used as substitutes for *Cannabis sativa*. Forensic cases of synthetic cannabinoid use have increased recently; the adverse effects of indazole-carboxamide-type synthetic cannabinoids, especially, present severe social problems [2].

In a previous study, we developed a method to separate enantiomers of synthetic cannabinoids by liquid chromatography–mass spectrometry (LC–MS) [3]. In the case of NPS classified into cathinone and phenethylamine groups, several reports have concluded that (*S*)-enantiomers are more potent than (*R*)-enantiomers [4–7]. Among the carboxamide-type synthetic cannabinoids, some compounds possess asymmetric carbon, which is derived from an amide moiety composed of amino acid derivatives (i.e., amides or esters of amino acids). These classes of compounds were first developed by Pfizer as potential therapeutic drugs; however, their patent only includes (*S*)-enantiomers, even though the structures of these compounds have chiral centers [8]. Some previous studies reported the cannabimimetic activities of the chiral synthetic cannabinoids, but they all investigated only the (*S*)-enantiomers, and thus the pharmaceutical activities of the (*R*)-enantiomers remain unknown [9].

In this study, we synthesized both enantiomers of *N*-(1-amino-3-methyl-1-oxobutan-2-yl)-1-(2-fluorobenzyl)-1*H*-indazole-3-carboxamide (AB-FUBINACA 2-fluorobenzyl isomer), *N*-(1-amino-1-oxo-3-phenylpropan-2-yl)-1-(cyclohexylmethyl)-1*H*-indazole-3-carboxamide (APP-CHMINACA), ethyl [1-(5-fluoropentyl)-1*H*-indazole-3-carbonyl]valinate (5F-EMB-PINACA), ethyl [1-(4-fluorobenzyl)-1*H*-indazole-3-carbonyl]valinate (EMB-FUBINACA), and methyl 2-[1-(4-fluorobenzyl)-1*H*-indole-3-carboxamido]-3,3-dimethylbutanoate (MDMB-FUBICA) to evaluate their potency to activate CB_1_ and CB_2_ cannabinoid receptors.

## Materials and methods

### Reagents

Methyl 1*H*-indazole-3-carboxylate and (bromomethyl)cyclohexane were purchased from Sigma-Aldrich (St. Louis, MO, USA); 1-bromo-5-fluoropentane from Fluorochem Ltd. (Hadfield, UK); d-valine ethyl ester hydrochloride, l-valine ethyl ester hydrochloride, and d-valine amide hydrochloride from Combi-Blocks Inc. (San Diego, CA, USA); d-phenylalanine amide hydrochloride from Watanabe Chemical Industries Ltd. (Hiroshima, Japan). All other reagents used in this study were purchased from Wako Pure Chemical Industries (Osaka, Japan).

### Nuclear magnetic resonance spectroscopy

Nuclear magnetic resonance (NMR) spectra were recorded using an ECZS-400 spectrometer (JEOL Resonance, Tokyo, Japan) with dimethyl sulfoxide (DMSO)-*d*_6_ as the solvent. The chemical shifts *δ* were recorded in ppm relative to tetramethylsilane (^1^H: *δ* = 0 ppm, ^13^C: *δ* = 0 ppm) or the solvent (^13^C: *δ* = 39.5 ppm) as an internal standard. The compounds were assigned by ^1^H NMR, ^13^C NMR, distortionless enhancement by polarization transfer, ^1^H-^13^C heteronuclear multiple quantum coherence, ^1^H-^13^C heteronuclear multiple-bond correlation, and ^1^H-^1^H correlation spectroscopy.

### Chiral chromatography

Chirality of the compounds was confirmed by LC–MS as described previously [3]. A CHIRALPAK AZ-3R column (3.0 μm particle size, 150 × 2.1 mm i.d.) (Daicel Corporation, Osaka, Japan) was used, and the mobile phase was composed of H_2_O/acetonitrile (55:45, v/v) under isocratic conditions. The flow rate of the mobile phase was 0.3 mL/min, and the injection volume was 1 μL. The column temperature was 40 °C. The stock standard solutions were prepared as described previously [3]. When necessary, the sample solutions and the stock standard solutions were diluted by the mobile phase for the liquid chromatography–high-resolution-mass spectrometry (LC–HR-MS) analysis [3].

### Chemical synthesis

**Fig. 1:**
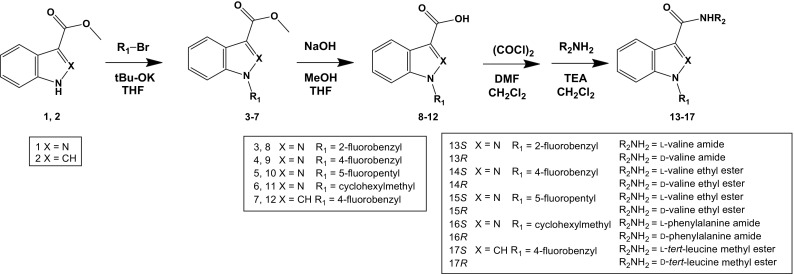
Procedures for synthesizing the enantiomers dealt with in this study. *tBu-OK* potassium *tert*-butoxide, *THF* tetrahydrofuran, *DMF N*,*N*-dimethylformamide, *(COCl)*_2_ oxalyl chloride, *TEA* triethylamine, *MeOH* methanol

**Fig. 2:**
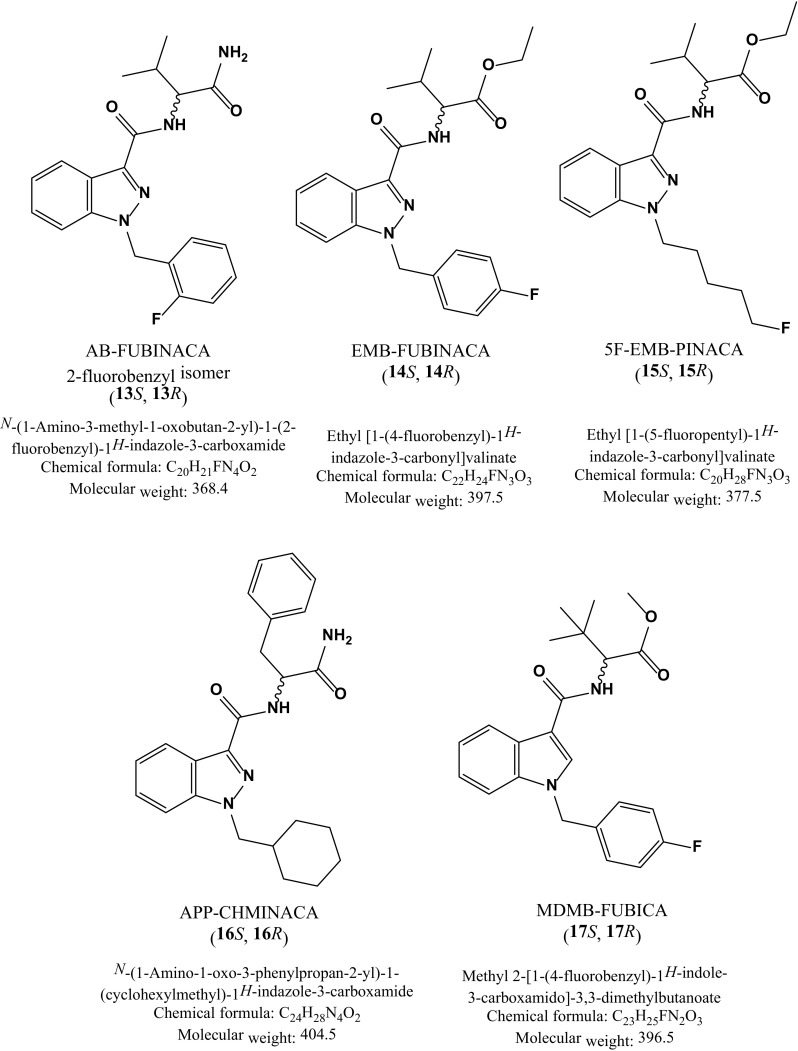
Chemical structures of the target compounds in this study

The synthetic pathway of these enantiomers is shown in Fig. 1. All of the target compounds were made from methyl 1*H*-indazole-3-carboxylate (compound **1**) or methyl 1*H*-indole-3-carboxylate (compound **2**). The enantiomers of the target compounds were synthesized by a slightly modified version of a previously described method [3, 10]. Compound **1** or **2** was *N*-alkylated by alkyl halide under basic conditions and yielded compounds that were deprotected by hydrolysis of the methyl group. The target compounds were synthesized by chlorination of the carboxylic acids by oxalyl chloride and then amidation with the amino acid derivatives. The chemical structures of the compounds dealt with in this study are shown in Fig. 2.

#### *N*-Alkylation of methyl 1*H*-indazole/indole-3-carboxylate

Compound **5** was synthesized as described previously [3]. Compounds **3**, **4**, **6**, and **7** were prepared in the same manner, except for the starting compounds. To a solution of compound **1** or **2** in tetrahydrofuran (THF), potassium *tert*-butoxide (*t*-BuOK) (1.2 equivalent) was added. To the flask containing the solution, alkyl halide (1.5 equivalent) was added and stirred at room temperature for more than 12 h. Ethyl acetate and distilled water were added to the solution, and the organic layer was separated from the aqueous layer. The organic layer was then washed with saturated aqueous NaCl and dried with anhydrous MgSO_4_. The solution was filtered, and the solvent was removed under a vacuum. The residue was purified by an Isolela Speckt System (Biotage, Tokyo, Japan) on a SNAP Ultra column (Biotage) with ethyl acetate in *n*-hexane as the mobile phase. The solvent of the obtained fraction was removed under a vacuum to yield compounds **3**, **4**, **5**, **6** or **7**.

#### Hydrolysis of methyl *N*-alkyl-1*H*-indazole-3-carboxylate

Synthesis of compound **10** was performed as described previously [3]. Compounds **8**, **9**, and **11** were synthesized similarly, except for the starting compounds. To a flask containing compounds **3**, **4**, or **6**, THF, ethanol, and 1 M NaOH_(aq)_ (approximately 2.5 mL/1 mmol of the material compounds) were added and stirred at room temperature for more than 16 h. After the reaction, the organic solvents were removed under a vacuum. Ethyl acetate and 20% Na_2_CO_3_ in H_2_O were added to the solution and stirred, and the solution was allowed to separate. The organic layer was extracted by 20% Na_2_CO_3_ in H_2_O again, and the aqueous layers were combined. The organic layer was extracted twice with 1 M NaOH in H_2_O. The aqueous layers were combined and neutralized with concentrated HCl, extracted with ethyl acetate twice, and then the organic layer was washed twice with brine. Anhydrous MgSO_4_ was added to the organic layer and removed under a vacuum to yield compounds **8**, **9**, or **11**.

#### Hydrolysis of methyl *N*-alkyl-1*H*-indole-3-carboxylate

Compound **12** was synthesized by the hydrolysis of compound **7**. To a flask containing compound **7**, THF, ethanol, methanol, and 10 M NaOH_(aq)_ (approximately 2 mL/1 mmol of compound **7**) were added and stirred at room temperature for 2.5 h. After the reaction, the organic solvents were removed under a vacuum. Ethyl acetate was added to the solution and mixed well, and the solution was allowed to separate. The organic layer was extracted twice with 1 M NaOH_(aq),_ and the aqueous layers were then combined. The aqueous layer was neutralized with HCl and extracted with ethyl acetate twice. The organic layer was then washed twice with brine. Subsequently, anhydrous MgSO_4_ was added to the combined organic layer and removed under a vacuum to yield compound **12**.

#### (*S*)-*N*-(1-Amino-3-methyl-1-oxobutan-2-yl)-1-(2-fluorobenzyl)-1*H*-indazole-3-carboxamide (**13***S*)

To a flask containing compound **8** in dichloromethane (20 mL/1 mmol of compound **8**), *N*,*N*-dimethylformamide (20 μL/1 mmol) and oxalyl chloride (2.5 equivalent) were added and stirred at room temperature for 30 min. The solvent was then removed under a vacuum to obtain 1-(2-fluorobenzyl)-1*H*-indazole-3-carbonyl chloride as a hazy oily residue. The residue was dissolved in dichloromethane (10 mL), and l-valine amide hydrochloride (1.1 equivalent) was added. Triethylamine (1 mmol) was added to the mixture and then the resulting solution was stirred at room temperature for 90 min. The solvent was removed under reduced pressure and dissolved in ethyl acetate. The solvent was separated with water and washed with 0.1 M HCl, saturated NaHCO_3_, and saturated NaCl. Drying with MgSO_4_ and removal of the solvent afforded compound **13** *S* as a fibrous white solid.

^1^H NMR (DMSO-*d*_6_): *δ* 8.19 (1H, dd, *J* = 8.0, 1.0 Hz), 7.77 (1H, d, *J* = 8.5 Hz), 7.69 (1H, d, *J* = 9.0 Hz), 7.65 (1H, s), 7.48 (1H, t-like), 7.43–7.34 (1H, m), 7.30 (1H, dd, *J* = 8.0, 7.0 Hz), 7.26–7.18 (2H, m), 7.17–7.09 (2H, m), 5.84 (2H, s), 4.42 (1H, dd, *J* = 9.0, 6.0 Hz), 2.09 (1H, m), 0.94 (3H, d, *J* = 7.0 Hz), 0.89 (3H, d, *J* = 7.0 Hz). ^13^C NMR (DMSO-*d*_6_): *δ* 172.5, 161.1, 159.9 (d, *J* = 246 Hz), 140.8, 137.2, 130.1 (d, *J* = 8 Hz), 129.7 (d, *J* = 4 Hz), 127.0, 124.7 (d, *J* = 4 Hz), 123.5 (d, *J* = 14 Hz), 122.7, 122.1, 121.8, 115.5 (d, *J* = 21 Hz), 110.4, 56.8, 46.5 (d, *J* = 3 Hz), 31.2, 19.3, 17.9.

#### (*R*)-*N*-(1-Amino-3-methyl-1-oxobutan-2-yl)-1-(2-fluorobenzyl)-1*H*-indazole-3-carboxamide (**13***R*)

Subjecting compound **8** to a procedure similar to that for **13** *S*, but substituting d-valine amide hydrochloride for l-valine amide, yielded **13** *R* as a fibrous white solid.

^1^H NMR (DMSO-*d*_6_): *δ* 8.20 (1H, d, *J* = 8.0 Hz), 7.77 (1H, d, *J* = 8.5 Hz), 7.71 (1H, d, *J* = 9.0 Hz), 7.66 (1H, s), 7.48 (1H, t-like), 7.42–7.34 (1H, m), 7.30 (1H, t-like), 7.28–7.20 (2H, m), 7.18–7.10 (2H, m), 5.84 (2H, s), 4.42 (1H, dd, *J* = 9.0, 6.0 Hz), 2.10 (1H, m), 0.94 (3H, d, *J* = 7.0 Hz), 0.89 (3H, d, *J* = 7.0 Hz). ^13^C NMR (DMSO-*d*_6_): *δ* 172.5, 161.2, 159.9 (d, *J* = 247 Hz), 140.8, 137.2, 130.2 (d, *J* = 9 Hz), 129.8 (d, *J* = 4 Hz), 127.0, 124.7 (d, *J* = 3 Hz), 123.6 (d, *J* = 15 Hz), 122.7, 122.1, 121.8, 115.6 (d, *J* = 21 Hz), 110.5, 56.8, 46.5 (d, *J* = 3 Hz), 31.2, 19.4, 18.0.

#### Ethyl [1-(4-fluorobenzyl)-1*H*-indazole-3-carbonyl]-l-valinate (**14***S*)

Subjecting compound **9** to a procedure similar to that for **13** *S*, but substituting l-valine ethyl ester hydrochloride for l-valine amide, yielded **14** *S* as a clear oily residue.

^1^H NMR (DMSO-*d*_6_): *δ* 8.15 (1H, d, *J* = 8.5 Hz), 8.12 (1H, d, *J* = 8.0 Hz), 7.80 (1H, d, *J* = 8.5 Hz), 7.47 (1H, ddd-like), 7.37–7.32 (2H, m), 7.30 (1H, t-like), 7.20–7.14 (2H, m), 5.78 (2H, s), 4.45 (1H, dd, *J* = 8.0, 7.0 Hz), 4.22–4.12 (2H, m), 2.30–2.22 (1H, m), 1.22 (3H, t, *J* = 7.0 Hz), 0.97 (6H, t-like). ^13^C NMR (DMSO-*d*_6_): *δ* 171.5, 161.9, 161.6 (d, *J* = 244 Hz), 140.5, 136.9, 133.0 (d, *J* = 3 Hz), 129.5 (d, *J* = 8 Hz), 127.0, 122.8, 122.4, 121.7, 115.5 (d, *J* = 21 Hz), 110.6, 60.6, 57.4, 51.6, 29.9, 19.0, 18.7, 14.1.

#### Ethyl [1-(4-fluorobenzyl)-1*H*-indazole-3-carbonyl]-d-valinate (**14***R*)

Subjecting compound **9** to a procedure similar to that for **13** *S*, but substituting d-valine ethyl ester hydrochloride for l-valine amide, yielded **14** *R* as a clear oily residue.

^1^H NMR (DMSO-*d*_6_): *δ* 8.15 (1H, d, *J* = 8.5 Hz), 8.12 (1H, d, *J* = 8.5 Hz), 7.80 (1H, d, *J* = 8.5 Hz), 7.46 (1H, ddd-like), 7.37–7.32 (2H, m), 7.29 (1H, t-like), 7.20–7.14 (2H, m), 5.78 (2H, s), 4.45 (1H, dd, *J* = 8.5, 6.5 Hz), 4.22–4.12 (2H, m), 2.30–2.22 (1H, m), 1.22 (3H, t, *J* = 7.0 Hz), 0.97 (6H, t-like). ^13^C NMR (DMSO-*d*_6_): *δ* 171.5, 161.8, 161.6 (d, *J* = 244 Hz), 140.5, 136.9, 133.0 (d, *J* = 3 Hz), 129.5 (d, *J* = 8 Hz), 127.0, 122.8, 122.4, 121.7, 115.5 (d, *J* = 21 Hz), 110.6, 60.6, 57.4, 51.6, 29.9, 19.0, 18.7, 14.1.

#### Ethyl [1-(5-fluoropentyl)-1*H*-indazole-3-carbonyl]-l-valinate (**15***S*)

Subjecting compound **10** to a procedure similar to that for **13** *S*, but substituting l-valine ethyl ester hydrochloride for l-valine amide, yielded **15** *S* as a clear oily residue.

^1^H NMR (DMSO-*d*_6_): *δ* 8.14 (1H, d, *J* = 8.0 Hz), 7.99 (1H, d, *J* = 8.5 Hz), 7.81 (1H, d, *J* = 8.5 Hz), 7.47 (1H, ddd-like), 7.29 (1H, t-like), 4.54 (2H, t, *J* = 7.0 Hz), 4.47 (1H, t, *J* = 6.0 Hz), 4.42 (1H, dd, *J* = 8.5, 6.5 Hz), 4.35 (1H, t, *J* = 6.0 Hz), 4.22–4.12 (2H, m), 2.31–2.21 (1H, m), 1.97–1.88 (2H, m), 1.75–1.61 (2H, m), 1.41–1.31 (2H, m), 1.22 (3H, t, *J* = 7.0 Hz), 0.98 (3H, d, *J* = 4.5 Hz), 0.96 (3H, d, *J* = 4.0 Hz). ^13^C NMR (DMSO-*d*_6_): *δ* 171.5, 161.9, 140.6, 136.2, 126.7, 122.6, 122.1, 121.6, 110.5, 83.6 (d, *J* = 162 Hz), 60.6, 57.2, 48.6, 30.0, 29.3 (d, *J* = 19 Hz), 29.0, 22.0 (d, *J* = 6 Hz), 19.0, 18.6, 14.1.

#### Ethyl [1-(5-fluoropentyl)-1*H*-indazole-3-carbonyl]-d-valinate (**15***R*)

Subjecting compound **10** to a procedure similar to that for **13** *S*, but substituting d-valine ethyl ester hydrochloride for l-valine amide, yielded **15** *R* as a clear oily residue.

^1^H NMR (DMSO-*d*_6_): *δ* 8.14 (1H, d, *J* = 7.5 Hz), 7.99 (1H, d, *J* = 8.0 Hz), 7.81 (1H, d, *J* = 8.5 Hz), 7.47 (1H, ddd-like), 7.29 (1H, t-like), 4.53 (2H, t, *J* = 7.0 Hz), 4.47 (1H, t, *J* = 6.0 Hz), 4.43 (1H, dd, *J* = 8.5, 6.5 Hz), 4.35 (1H, t, *J* = 6.0 Hz), 4.24–4.10 (2H, m), 2.31–2.20 (1H, m), 1.97–1.88 (2H, m), 1.75–1.61 (2H, m), 1.41–1.31 (2H, m), 1.22 (3H, t, *J* = 7.0 Hz), 0.98 (3H, d, *J* = 4.0 Hz), 0.96 (3H, d, *J* = 4.5 Hz). ^13^C NMR (DMSO-*d*_6_): *δ* 171.5, 161.9, 140.6, 136.2, 126.7, 122.6, 122.1, 121.6, 110.5, 83.6 (d, *J* = 162 Hz), 60.6, 57.2, 48.6, 30.0, 29.3 (d, *J* = 19 Hz), 29.0, 22.0 (d, *J* = 6 Hz), 19.0, 18.6, 14.1.

#### (*S*)-*N*-(1-Amino-1-oxo-3-phenylpropan-2-yl)-1-(cyclohexylmethyl)-1*H*-indazole-3-carboxamide (**16***S*)

Subjecting **11** to a procedure similar to that for **13** *S*, but substituting l-phenylalanine amide hydrochloride for l-valine amide yielded **16** *S* as a crystalline white solid.

^1^H NMR (DMSO-*d*_6_): *δ* 8.11 (1H, d, *J* = 8.0 Hz), 7.90 (1H, d, *J* = 8.0 Hz), 7.76 (1H, d, *J* = 8.5 Hz), 7.67 (1H, s), 7.45–7.41 (1H, m), 7.26–7.14 (7H, m), 4.78–4.72 (1H, m), 4.37–4.26 (2H, m), 3.19–3.05 (2H, m), 1.99–1.87 (1H, m), 1.64–1.60 (3H, m), 1.47 (2H, t-like), 1.23–1.10 (3H, m), 1.07–0.97 (2H, m). ^13^C NMR (DMSO-*d*_6_): *δ* 172.6, 161.3, 141.1, 137.6, 136.4, 129.3, 128.0, 126.5, 126.3, 122.4, 121.8, 121.6, 110.6, 54.5, 53.2, 38.4, 37.7, 30.0, 25.8, 25.2, 25.1.

#### (*R*)-*N*-(1-Amino-1-oxo-3-phenylpropan-2-yl)-1-(cyclohexylmethyl)-1*H*-indazole-3-carboxamide (**16***R*)

Subjecting compound **11** to a procedure similar to that for **16** *S*, but substituting d-phenylalanine amide hydrochloride for l-phenylalanine amide hydrochloride, yielded **16** *R* as a crystalline white solid.

^1^H NMR (DMSO-*d*_6_): *δ* 8.11 (1H, d, *J* = 8.0 Hz), 7.90 (1H, d, *J* = 8.0 Hz), 7.76 (1H, d, *J* = 8.5 Hz), 7.66 (1H, s), 7.45–7.41 (1H, m), 7.26–7.14 (7H, m), 4.78–4.72 (1H, m), 4.37–4.26 (2H, m), 3.19–3.05 (2H, m), 1.97–1.88 (1H, m), 1.64–1.60 (3H, m), 1.47 (2H, t-like), 1.24–1.10 (3H, m), 1.07–0.97 (2H, m). ^13^C NMR (DMSO-*d*_6_): *δ* 172.6, 161.3, 141.1, 137.6, 136.4, 129.3, 128.0, 126.5, 126.3, 122.3, 121.8, 121.6, 110.6, 54.5, 53.2, 38.4, 37.7, 30.0, 25.8, 25.2, 25.1.

#### Methyl (*S*)-2-[1-(4-fluorobenzyl)-1*H*-indole-3-carboxamido]-3,3-dimethylbutanoate (**17***S*)

Subjecting **12** to a procedure similar to that for **13** *S*, but substituting l-*tert*-leucine methyl ester hydrochloride for l-valine amide yielded **17** *S* as a white powder.

^1^H NMR (DMSO-*d*_6_): *δ* 8.50 (1H, s), 8.11 (1H, d, *J* = 8.0 Hz), 7.71 (1H, d, *J* = 8.5 Hz), 7.52 (1H, d, *J* = 8.5 Hz), 7.34–7.31 (2H, m), 7.20–7.11 (4H, m, overlapped), 5.47 (2H, s), 4.50 (1H, d, *J* = 8.5 Hz), 3.66 (3H, s), 1.04 (9H, s). ^13^C NMR (DMSO-*d*_6_): *δ* 172.0, 164.3, 161.6 (d, *J* = 244 Hz), 136.0, 133.8 (d, *J* = 3 Hz), 132.1, 129.2 (d, *J* = 8 Hz), 126.9, 122.2, 121.3, 120.9, 115.5 (d, *J* = 22 Hz), 110.6, 109.3, 60.0, 51.4, 48.8, 33.8, 26.8.

#### Methyl (*R*)-2-[1-(4-fluorobenzyl)-1*H*-indole-3-carboxamido]-3,3-dimethylbutanoate (**17***R*)

Subjecting compound **12** to a procedure similar to that for **17** *S*, but substituting d-*tert*-leucine methyl ester hydrochloride for l-*tert*-leucine methyl ester hydrochloride, yielded **17** *R* as a white powder.

^1^H NMR (DMSO-*d*_6_): *δ* 8.50 (1H, s), 8.11 (1H, d, *J* = 7.0 Hz), 7.71 (1H, d, *J* = 8.5 Hz), 7.53 (1H, d, *J* = 8.0 Hz), 7.34–7.31 (2H, m), 7.20–7.13 (4H, m, overlapped), 5.47 (2H, s), 4.50 (1H, d, *J* = 8.5 Hz), 3.66 (3H, s), 1.04 (9H, s). ^13^C NMR (DMSO-*d*_6_): *δ* 172.0, 164.3, 161.6 (d, *J* = 244 Hz), 136.0, 133.7 (d, *J* = 3 Hz), 132.1, 129.2 (d, *J* = 8 Hz), 126.9, 122.2, 121.2, 120.9, 115.5 (d, *J* = 21 Hz), 110.6, 109.3, 60.0, 51.4, 48.7, 33.8, 26.8.

### In vitro assays to evaluate the CB_1_/CB_2_ receptor activities

For the evaluation of CB_1_/CB_2_ cannabinoid receptor activity, [^35^S]GTPγS binding assays were performed. These assays were performed at ADME and Tox. Research Institute, Sekisui Medical Co., Ltd. (Tokai-mura, Ibaraki, Japan). The assay conditions were as described previously [11], except for the tested concentration levels of the compounds ranging from 1 × 10^−11^ to 1 × 10^−4^ M. Agonistic activities (EC_50_ value: concentration showing 50% response) of the test compound to the cannabinoid receptors CB_1_ and CB_2_ were measured.

## Results and discussion

In this study, we synthesized both (*S*)- and (*R*)-enantiomers of synthetic cannabinoids to evaluate their pharmacological properties. All target compounds were reported in an EMCDDA-Europol joint report to be NPS sold and recreationally used in Europe [12, 13]. NMR data for the enantiomers were similar, as shown in the section on materials and methods above, and the results are in good agreement with the values expected for their structures.

**Fig. 3:**
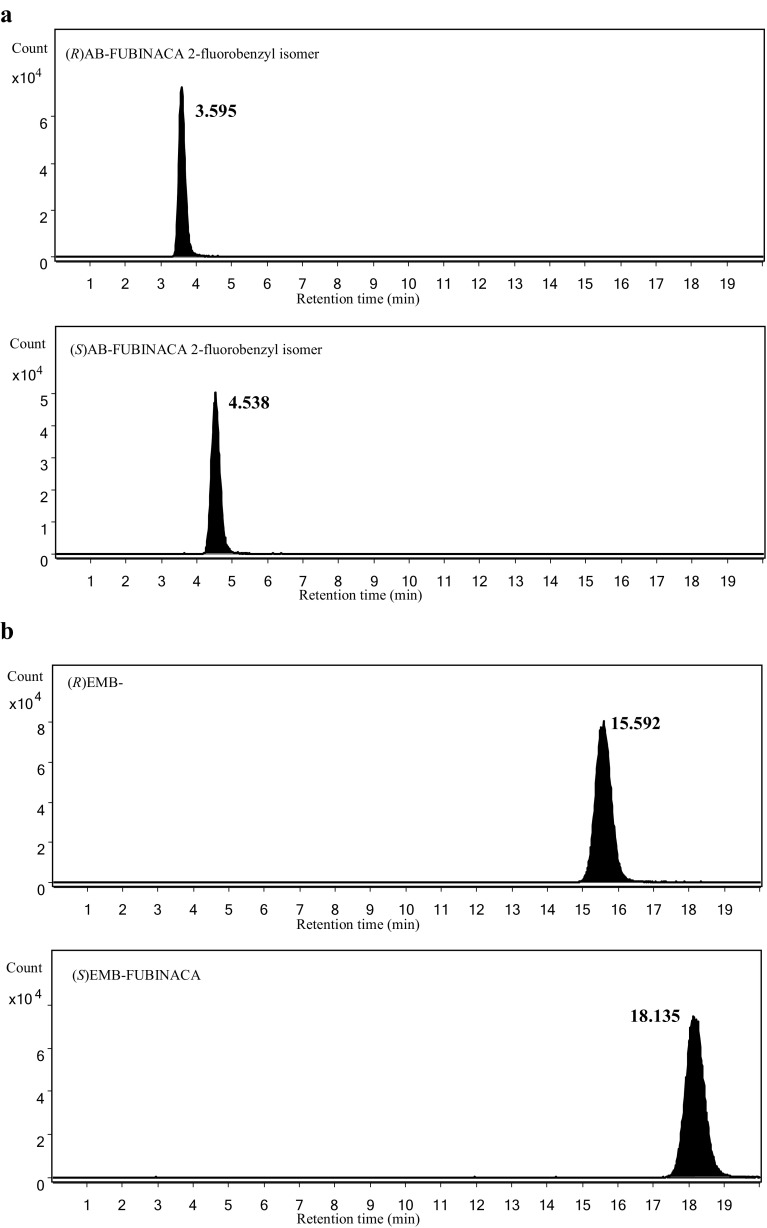

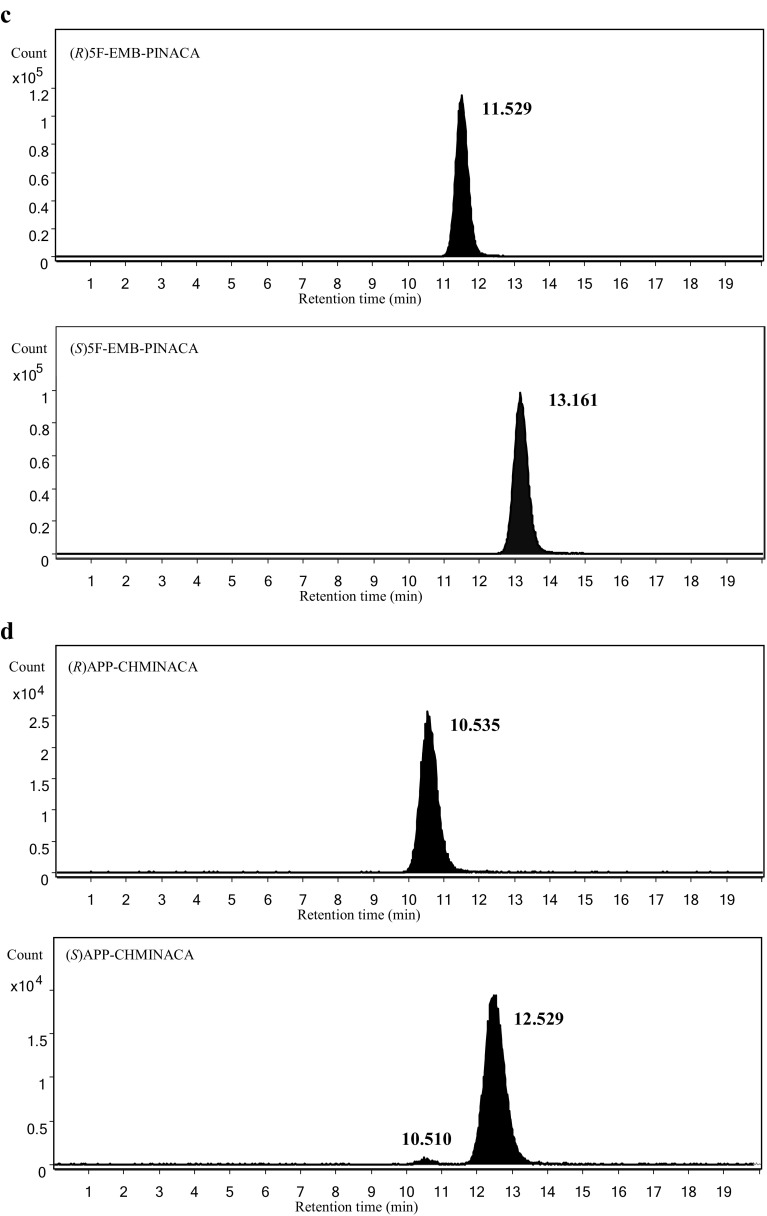

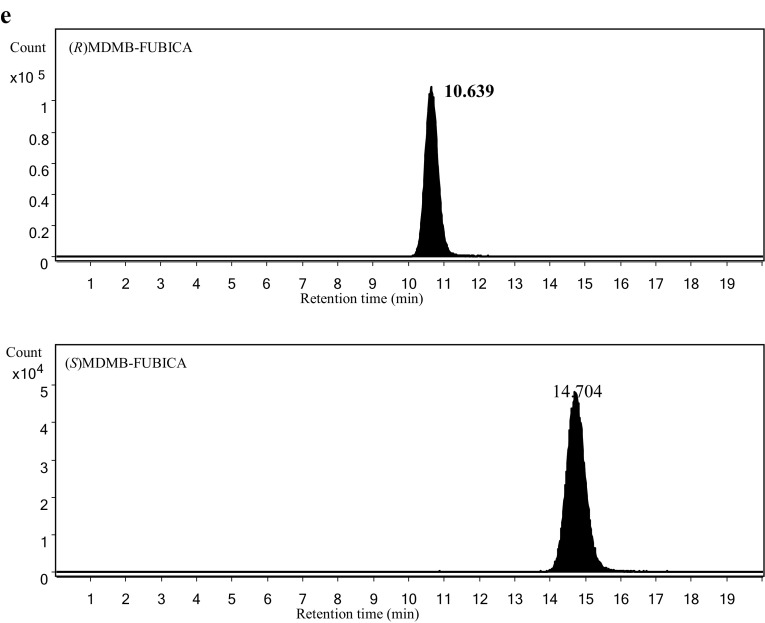
Extracted ion chromatograms of **a** AB-FUBINACA 2-fluorobenzyl isomer at *m/z* 369.1721 (Δ = ±0.5 mDa), **b** EMB-FUBINACA at *m/z* 398.1874 (Δ = ±0.5 mDa), **c** 5F-EMB-FUBINACA at *m/z* 378.2187 (Δ = ±0.5 mDa), **d** APP-CHMINACA at *m/z* 405.2285 (Δ = ±0.5 mDa), and **e** MDMB-FUBICA at *m/z* 397.1922 (Δ = ±0.5 mDa), as a function of enantiomers, obtained by liquid chromatography–high-resolution mass spectrometry

Figure 3 shows the extracted ion chromatograms for the enantiomers of the target compounds (1 μg/mL solution for both enantiomers) obtained by LC–HR-MS using the method that we developed previously [3]. By the previously developed method, all of the enantiomers of the tested compounds were separated, and no enantio-impurities were found in their chromatograms, except for (*S*) APP-CHMINACA. Approximately less than 2% of (*R*) APP-CHMINACA was confirmed in the extracted ion chromatogram of (*S*) APP-CHMINACA as shown in Fig. 3. However, because (*R*)-enantiomer was much less effective than (*S*)-enantiomer to both CB_1_ and CB_2_ receptors, it is suspected that the effect of contaminated (*R*)-enantiomer on the EC_50_ value was almost negligible.

Table 1 shows the results of the [^35^S]GTPγS binding assays performed in this study. Each compound functioned as both a CB_1_ and a CB_2_ receptor agonist, except for (*R*) MDMB-FUBICA. The EC_50_ values of (*S*)-enantiomers for the CB_1_ receptors were at least one-fifth of or less than those of (*R*)-enantiomers, which indicates that (*S*)-enantiomers are more potent CB_1_ receptor agonists than (*R*)-enantiomers. The (*S*)-enantiomer of APP-CHMINACA could activate CB_1_ at a concentration 134 times lower than that for the (*R*)-enantiomer. For MDMB-FUBICA, the CB_1_ receptor activity was not confirmed for the (*R*)-enantiomer even at the maximum tested concentration, but the EC_50_ of the (*S*)-enantiomer was 9.72 × 10^−9^ M; the difference in the EC_50_ levels between the enantiomers of MDMB-FUBICA to activate the CB_1_ receptor was more than 10,000-fold, which indicates that the activities for the CB_1_ receptor are markedly different depending on the chirality of the compounds. In contrast, we could not observe a clear relevance between EC_50_ values for the CB_2_ receptor and chirality of the compounds. (*R*) APP-CHMINACA and (*R*) MDMB-FUBICA lacked the potential to be CB_1_ receptor agonists, but both of their EC_50_ values for the CB_2_ receptor were less than 10^−6^ M; all of the target compounds were strong agonists for the CB_2_ receptor, irrespective of their chirality.

**Table 1:** Activities of enantiomers of each compound at human CB_1_ and CB_2_ receptors

Compound name	Human CB_1_ receptor		Human CB_2_ receptor		Selective index^b^ (CB_1_/CB_2_)
	EC_50_ (M)	*S*/*R* ratio^a^	EC_50_ (M)	*S*/*R* ratio^a^	
(*S*)AB-FUBINACA 2-fluorobenzyl isomer	2.92 × 10^−9^	0.21	2.44 × 10^−9^	3.9	1.20
(*R*)AB-FUBINACA 2-fluorobenzyl isomer	1.41 × 10^−8^		6.27 × 10^−10^		22.5
(*S*)APP-CHMINACA	2.51 × 10^−7^	0.0074	8.09 × 10^−9^	0.017	31.0
(*R*)APP-CHMINACA	3.37 × 10^−5^		4.90 × 10^−7^		68.8
(*S*)EMB-FUBINACA	4.58 × 10^−10^	0.015	2.14 × 10^−9^	2.5	0.214
(*R*)EMB-FUBINACA	3.07 × 10^−8^		8.63 × 10^−10^		35.6
(*S*)5F-EMB-PINACA	4.96 × 10^−9^	0.14	6.91 × 10^−9^	4.1	0.718
(*R*)5F-EMB-PINACA	3.59 × 10^−8^		1.68 × 10^−9^		21.4
(*S*)MDMB-FUBICA	9.72 × 10^−9^	<0.0001	1.07 × 10^−9^	0.35	9.08
(*R*)MDMB-FUBICA	>1.00 × 10^−4^		3.10 × 10^−9^		>30,000
CP55940	1.28 × 10^−9^		1.54 × 10^−10^		8.3

^a^ *S*/*R* ratio is the EC_50_ value ratio of an (*S*)-enantiomer to the corresponding (*R*)-enantiomer

^b^Selective index is EC_50_ ratio of the CB_1_ to CB_2_

As for the CB_1_/CB_2_ selectivity, (*R*)-enantiomers tended to be CB_2_-selective and were at least 20-fold more active than for CB_1_ for all of the tested compounds. Interestingly, (*R*) MDMB-FUBICA functioned only on the CB_2_ receptor as an agonist, while showing no CB_1_ activity as mentioned above, although (*S*) MDMB-FUBICA could activate both of these receptors at nanomolar levels. In several preclinical studies, compounds such as JWH-133 and HU308 were used as CB_2_-selective ligands [14]. More recently, the synthesis and evaluation of novel CB_2_-selective agonists have been repeatedly reported. In these pharmacological or medicinal chemistry studies seeking CB_2_ agonists, the most selective compound showed a selective index of CB_1_/CB_2_ of only 100- to 1000-fold. In this study, (*R*) MDMB-FUBICA was shown to have potency to activate the CB_2_ receptor signal with an EC_50_ value of 3.1 × 10^−9^ M, but had no [^35^S]GTPγS binding activity for the CB_1_ receptor, even at 1.0 × 10^−4^ M, which corresponds to a selective index of more than 3.2 × 10^4^-fold.

The metabolism of MDMB-FUBICA enantiomers has not been reported yet; thus it remains unclear whether (*R*) MDMB-FUBICA can mimic the function of the CB_2_ receptor agonist when applied to an animal model to evaluate the effect on the central nervous system. Recently, it was reported that the (*S*)-enantiomer was mainly detected in herbal products containing chiral carboxamide-type synthetic cannabinoids [3, 15]. The cannabimimetic activities of each enantiomer presented in this study may be helpful to precisely recognize forensic cases associated with these synthetic cannabinoids when evaluated with the quantitative data of a specimen analyzed after enantiomeric separation.

## Conclusions

The activities of the enantiomers of five synthetic cannabinoids (AB-FUBINACA 2-fluorobenzyl isomer, APP-CHMINACA, 5F-EMB-PINACA, EMB-FUBINACA, and MDMB-FUBICA) were evaluated by the [^35^S]GTPγS binding assay. We also confirmed that these enantiomers could be clearly differentiated by chiral-LC–MS. These findings should contribute to better understanding of the forensic cases associated with these synthetic cannabinoids.
